# Detection of features predictive of microRNA targets by integration of network data

**DOI:** 10.1371/journal.pone.0269731

**Published:** 2022-06-09

**Authors:** Mert Cihan, Miguel A. Andrade-Navarro

**Affiliations:** Faculty of Biology, Johannes Gutenberg University, Biozentrum I, Mainz, Germany; Southwest University, CHINA

## Abstract

Gene activity is controlled by multiple molecular mechanisms, for instance through transcription factors or by microRNAs (miRNAs), among others. Established bioinformatics tools for the prediction of miRNA target genes face the challenge of ensuring accuracy, due to high false positive rates. Further, these tools present poor overlap. However, we demonstrated that it is possible to filter good predictions of miRNA targets from the bulk of all predictions by using information from the gene regulatory network. Here, we take advantage of this strategy that selects a large subset of predicted microRNA binding sites as more likely to possess less false-positives because of their over-representation in RE1 silencing transcription factor (REST)-regulated genes from the background of TargetScanHuman 7.2 predictions to identify useful features for the prediction of microRNA targets. These enriched miRNA families would have silencing activity for neural transcripts overlapping the repressive activity on neural genes of REST. We analyze properties of associated microRNA binding sites and contrast the outcome to the background. We found that the selected subset presents significant differences respect to the background: (i) lower GC-content in the vicinity of the predicted miRNA binding site, (ii) more target genes with multiple identical microRNA binding sites and (iii) a higher density of predicted microRNA binding sites close to the 3’ terminal end of the 3’-UTR. These results suggest that network selection of miRNA-mRNA pairs could provide useful features to improve microRNA target prediction.

## 1. Introduction

Post-transcriptional repression of mRNAs by microRNAs (miRNAs) is one of multiple layers of regulation of gene expression [1]. Since the discovery of the first miRNA, lin-4 in *Caenorhabditis elegans* in 1993 [2], more than 2,300 human miRNAs with numerous regulatory functions have been identified [3]. Particularly, the malfunctioning of miRNA regulation has been described as promoting neurological diseases [4] and various types of cancer [5], among other illnesses [6].

Estimates suggest that approximately 60% of protein-coding genes in the human genome may be regulated by miRNAs [7]. However, miRNA functional characterization is experimentally difficult due to their regulatory mechanisms, which are more subtle and less specific than transcription factors and epigenetic modifications [8]. This has fueled the development of many bioinformatics tools for the prediction of miRNA-mRNA interactions [9].

A commonly applied algorithm for detecting miRNA-target genes is based on finding 3’-UTR sequences with conserved sites that are complementary to the seed region of broadly conserved miRNAs, following the rules of Watson-Crick base pairing. Along with the integration of further criteria, such as the conservation of the 3’-UTR across mammalian species, the presence of complementary sequences around the matching seed and the assignment of binding free energy, many tools perform ranking of predicted miRNA-mRNA interactions to determine the probability of conserved targeting [10–13].

Regardless of these efforts, and although the regulatory mechanisms and biogenesis of miRNAs are well studied, the computational prediction of target genes and binding sites faces the challenge of ensuring accuracy due to false positive rates reaching 70% [14] and established predictors and databases such as TargetScan, miRanda and miRBase demonstrate poor agreement [15]. The lack of large collections of validated miRNA-mRNA interactions hampers the improvement of methods to predict these interactions.

Although transcription factors perform activity on the pre-transcriptional level and miRNAs on the post-transcriptional level, their systematics and effects exhibit a strong resemblance. Transcription factors and miRNAs are crucial components of the gene regulatory network which operate as trans-acting factors by interaction with cis-regulatory elements in the target gene [16]. The coordinated action of cell- and tissue-specific sets of transcription factors with multiple cis-regulatory elements controls development and often determines cell identity. Furthermore, many miRNAs are described as being exclusively present in specific cell types and having related functions [16]. Moreover, the 3’-UTRs of target genes are capable of possessing multiple cis-regulatory elements for distant miRNAs, indicating cluster-wise regulation and coordinated gene repression [16, 17]. Notably, coding genes for transcription factors and miRNAs regulate each other in feedback and feedforward loops, hinting at their interaction in a gene regulatory network [16, 18], and pairs of transcription factors and miRNAs coregulating common targets have been noted [19].

Previously, we analyzed the overlap between targets of transcription factors and targets of miRNAs for the purpose of identifying redundancy in the global regulatory network and to add support to large subsets of predicted miRNA-mRNA interactions [20]. Potential target genes for RE1 silencing transcription factor (REST) were identified by the analysis of multiple ChIP-seq datasets for diverse human cell types [20]. The selected transcription factor REST has been found to exert biological activity by regulating genes associated with abundant neuronal but also non-neuronal functions [21]. From the background of all miRNA-mRNA interactions predicted by TargetScanHuman 6.2 [12], we found 20 broadly conserved miRNA families (REST miRNAs) whose targets were over-represented in genes potentially regulated by REST. Several of these REST miRNAs had been previously described as contributing to neural cell differentiation and tumor suppression in glioblastoma. One of the REST miRNA-mRNA interactions with the highest support, miRNA-448 with the oncogene PI3KR1, was experimentally validated in our original work [20], and recent work found reported further effects of this miRNA in the regulation of the PI3K/AKT signaling pathway through targeting of ROCK1, inhibiting the progression of retinoblastoma [22].

Under the assumption that the predicted interactions between REST miRNAs and mRNAs are more accurate than other predictions, we propose that the study of the differences between these targets and the background of all predicted miRNA-mRNA interactions will point to features characterizing real interactions. Our aim is to discover target features that could be used to improve miRNA target prediction. We particularly focus on the analysis of the properties of miRNA binding sites in the 3’-UTR, thus attempting to reveal new features for factor-associated miRNA predictions. For this purpose, we study the position of miRNA binding sites, the presence of multiple targets, and the GC-content around the seed matching sequence in the 3’-UTR. Considering that the genetic locus as well as the combinatorial activity [17] and the accessibility of cis-regulatory elements [23], play a crucial role in transcriptional regulation, we assume that these features could also be important for miRNA regulation and could contribute to the improvement of the prediction of conserved miRNA targeting.

In our study, we compute the over-representation of REST miRNAs for miRNA-mRNA interactions predicted with the most recent version of TargetScanHuman (version 7.2), which extends the previous prediction model by considering several additional features when scoring predicted interactions, including the structural accessibility of the miRNA binding site, global and local nucleotide composition and 3’-UTR length [24].

## 2. Results

We analyzed properties of predicted miRNA binding sites for miRNA families targeting sets of genes enriched in genes that are potentially bound by the transcription factor REST (S2 Table), in terms of their position and nucleic environment. For simplicity, hereafter we name these families as REST miRNAs, the genes predicted to be bound by REST (or their 3’-UTRs) as REST genes (or 3’-UTRs), and the binding sites pairing REST miRNAs and their targets as REST pairs. We then contrasted the outcome to predicted miRNA-mRNA interactions for all human genes, as annotated by TargetScanHuman 7.2 (see Materials and Methods section for details). Conversely, we name the set of miRNAs as TargetScanHuman (TSH) miRNAs, the 3’-UTRs/genes predicted to be bound by TSH miRNAs as TSH 3’-UTRs/genes, and the binding sites pairing TSH miRNAs and their targets as TSH pairs. Table 1 presents an overview of descriptive statistics and calculated p-values for each analysis.

**Table 1:** Descriptive statistics and p-values for parsed properties of REST-associated miRNA-target gene pairs (REST) and TargetScanHuman miRNA-target gene pairs (TSH) or TargetScanHuman miRNA-target gene pairs in REST 3’-UTRs (TSH-REST) (see Materials and Methods for details).

Analysis	Dataset	N	Mean	Std. deviation	p-value
3’-UTR length	REST	2781	4635 nt	3377 nt	<0.001
	TSH	12989	2482 nt	2098 nt	
Distance from 3’-UTR start to miRNA binding site	REST	17325	1979 nt	2480 nt	<0.001
	TSH	103467	1648 nt	1926 nt	
Distance from miRNA binding site to 3’-UTR end	REST	17325	2650 nt	2713 nt	<0.001
	TSH	103467	2226 nt	2261 nt	
Position of miRNA binding site (relative)	REST	17325	0.437	0.325	0.012
	TSH	103467	0.444	0.323	
GC-content of 3’-UTRs	REST	2781	0.390	0.069	<0.001
	TSH	12989	0.441	0.095	
Distance between multiple miRNA binding sites	REST	2689	1886 nt	2978 nt	<0.001
	TSH (REST 3’-UTRs)	25946	1932 nt	2594 nt	<0.001
	TSH	11127	1451 nt	2014 nt	
GC-content between multiple miRNA binding sites	REST	2689	0.363	0.087	<0.001
	TSH (REST 3’-UTRs)	25946	0.391	0.094	<0.001
	TSH	11127	0.402	0.108	

### 2.1 3’-UTR length

**Fig 1:**
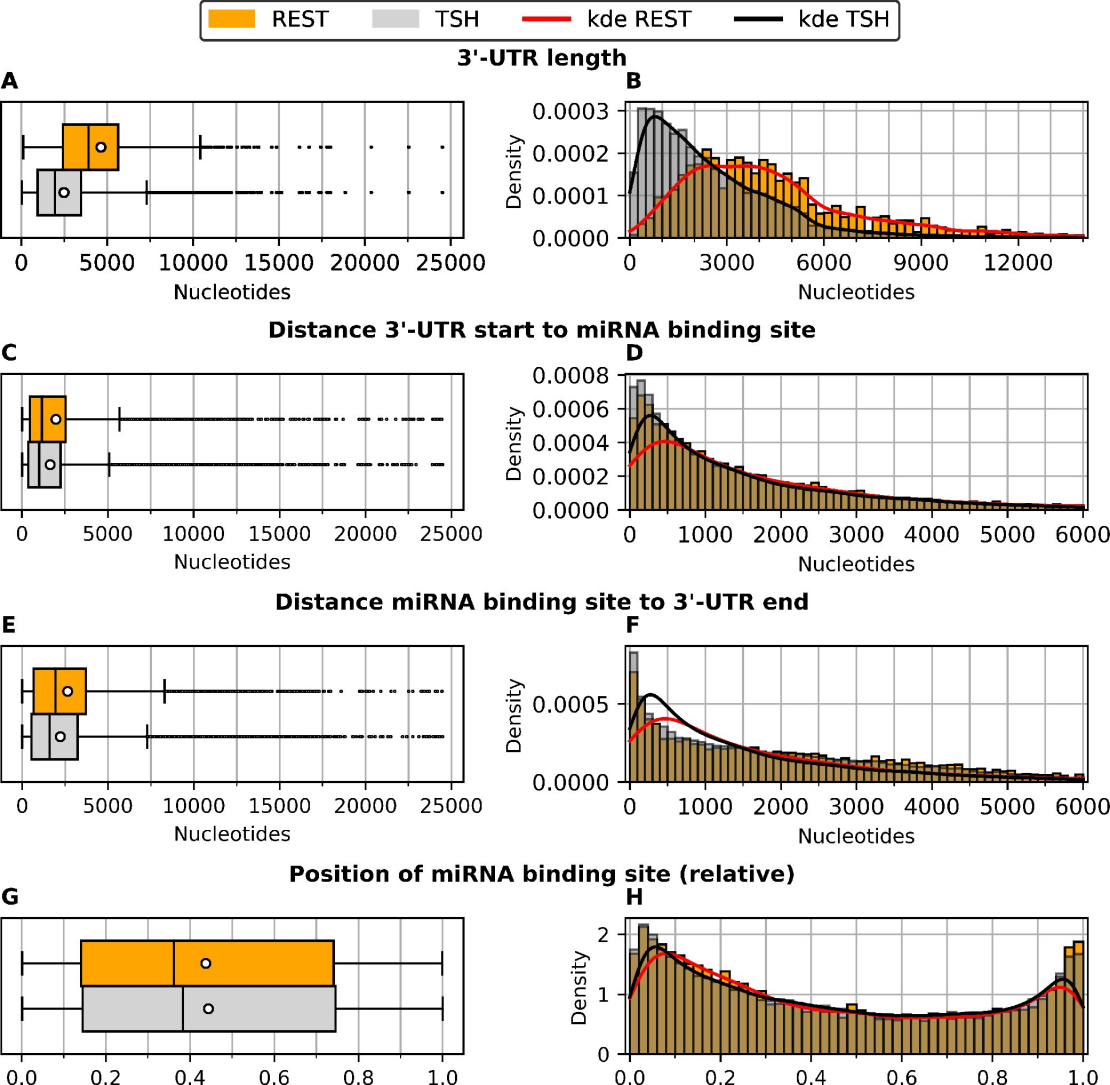
Properties of REST-associated miRNA-target gene pairs (REST) and TargetScanHuman miRNA-target gene pairs (TSH) or TargetScanHuman miRNA-target gene pairs in REST 3’-UTRs (TSH-REST). (A, B) Distribution of 3’-UTR length. (C, D) Distance from 3’-UTR start to miRNA binding site. (E, F) Distance from miRNA binding site to 3’-UTR end. (G, H) Relative position of miRNA binding site to the 3’-UTR length. Left side: the box plots indicate median, second and third quartile, mean (white dot) and standard deviation (whiskers). Right side: kde = kernel density of the corresponding distribution.

Predictions for REST pairs cover 2,781 target genes and are compared with 12,989 target genes for TSH pairs. The mean length for the 3’-UTR of REST-bound genes is 4,635 nt, which is 1.87-fold greater than for TargetScanHuman genes with an average length of 2,482 nt. The calculated p-value of <0.001 indicates the statistical significance of the difference in these means (Table 1). Both sets of genes present noticeably higher mean than median values, since they exhibit numerous outliers (Fig 1A). The histograms for REST-bound genes particularly display a higher density for 3’-UTRs longer than 3,000 nt and a lower density for 3’-UTRs shorter than 2,200 nt (Fig 1B). The plot of the distribution supports the statistical assessment that the subset of REST-bound genes with predicted miRNA binding sites have longer 3’-UTRs than the remaining annotated TargetScanHuman genes. We take this difference in consideration for the interpretation of the results of our further analyses.

### 2.2 Position of miRNA binding site

We measured the distance from the 3’ and 5’ terminal end of the 3’-UTR to the predicted miRNA binding site, as well as the relative miRNA binding site position in the 3’-UTR, for TSH pairs and REST pairs. We found that the mean of the absolute distance from the 5’ terminal end to the predicted miRNA binding site is significantly higher for REST pairs, with a p-value of <0.001 (Table 1; mean distances 1,979 nt and 1,648 nt, respectively) consistently with the significantly longer length of the 3’-UTRs of REST-bound genes. The distributions, however, display a strong resemblance (Fig 1C and 1D).

Similarly, the mean of the absolute distance from the predicted miRNA binding site to the 3’ terminal end of the 3’-UTR is significantly higher for REST pairs, with a p-value of <0.001 (Table 1; mean distances 2,650 nt and 2,226 nt, respectively) consistently with the significantly longer length of the 3’-UTRs of REST-bound genes. Again, however, the distributions present strong similarities (Fig 1E and 1F).

To obtain results independent of the length of the 3’-UTR, we calculated the position of the miRNA binding site relative to the length of the associated target 3’-UTR. The result shows that miRNA binding sites in both REST pairs and TSH pairs are located on average closer to the 5’ terminal end of the 3’-UTR. The mean position of miRNA binding sites for REST pairs is 0.437 and for TSH pairs 0.444. The statistical test yielded a p-value of 0.012, indicating that there were no significant differences for these means (Table 1). This result is consistent with the graphical representations of the distributions, which present strong similarities (Fig 1G and 1H).

All in all, miRNA binding sites for REST pairs are observed to have a greater absolute distance to the 5’ and 3’ terminal end of their 3’-UTRs, consistent with the longer length of observed REST 3’-UTRs, as well as a very similar position, relative to the 3’-UTR length.

### 2.3 GC-content around predicted miRNA biding sites

**Fig 2:**
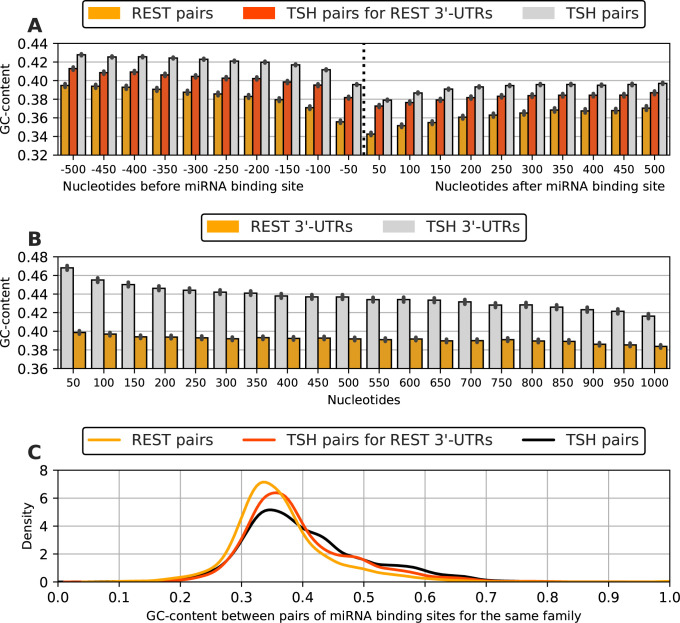
GC-content in the vicinity of predicted miRNA binding for subsets of miRNA-mRNA pairs. (A) GC-content relative to the distance around the miRNA binding site for REST pairs, TSH pairs and TSH pairs in the 3’-UTRs of REST regulated genes (TSH pairs in REST 3’-UTRs). (B) Background GC content for REST 3’-UTRs and TSH 3’-UTRs (see Methods for details). The error bars indicate the 99% confidence interval. (C) Kernel density estimation for the distribution of GC-content values between pairs of miRNA binding sites for the same miRNA family. Values obtained from TSH pairs, TSH pairs in REST 3’-UTRs and REST pairs.

The GC-content around predicted miRNA binding sites was calculated for 50 nt bins in the range 500 nt before and after the site (Fig 2A). This analysis revealed a notable decrease in GC-content towards the predicted miRNA binding site for all subsets, which was more marked in the REST miRNA-target pairs than in TSH miRNA-pairs (Fig 2A; orange and gray bars, respectively). However, the differences in the leftmost and rightmost values suggested that REST 3’-UTRs have a lower GC background content and, additionally, that the GC background content has a decreasing gradient that must be appreciable in a 1000 nt region. To test this hypothesis, we computed these backgrounds using 1000 nt regions taken at random positions from all REST 3’-UTRs and from all TSH 3’-UTRs, respectively. We obtained the expected results (lower GC content in REST 3’-UTRs and decreasing values from 5’ to 3’; Fig 2B).

To test that the differences in GC content variation surrounding REST miRNA and TSH miRNA pairs are not just due to differences in 3’-UTR properties, we examined separately the GC-content surrounding TSH miRNA pairs in REST 3’-UTRs (red bars in Fig 2A) and confirmed that their decrease in GC contents is also less pronounced than that for REST miRNA pairs.

Interestingly, we observed the largest drop in GC-content in the 50 nt bin right after the miRNA binding site, which could be a property used to improve miRNA predictions.

### 2.4 Presence of multiple predicted binding sites of a given miRNA family

Many 3’-UTRs possess multiple miRNA binding sites for the same miRNA family. For the set of TSH pairs, we identified 7,871 miRNA-target gene combinations with multiple miRNA binding sites within the target 3’-UTR. Furthermore, we observed 1,855 out of 15,009 miRNA-target gene pairs with more than one predicted binding site of the same miRNA family in the set of predicted REST pairs. In fact, all 67 miRNA families that are enriched in the subset of REST regulated genes have multiple binding sites in the 3’-UTRs of the target genes.

p-values were calculated using the Fisher’s exact test, to test for statistically significant differences in the proportions of target genes with at least one predicted binding site and target genes with multiple binding sites, between REST pairs and TSH pairs. The analysis returned a p-value of <0.001, concluding statistical significance (S4 Table). We also computed p-values for the proportion of multiple binding sites for each miRNA family enriched for REST-bound genes, compared to the observation of TSH pairs. A total of 36 out of 67 enriched miRNA families present a statistically significant difference (p-value <0.05) for the proportion of target genes with multiple miRNA binding sites for the same family, compared to background predictions for all considered miRNA-mRNA interactions. Remarkably, the 21 REST miRNA families with the largest number of associated miRNA-target gene pairs (277 or more) show significance (S4 Table). This suggests that the presence of multiple miRNA binding sites for the same family is a good predictor of miRNA binding sites.

### 2.5 Distance and GC-content between multiple predicted binding sites

We further examined properties from previously identified multiple binding sites for a given miRNA family in 3’-UTRs, in terms of distance and GC-content. The mean of the distance between multiple miRNA binding sites for the subset of REST pairs is 1,886 nt, whereas the mean for TSH predictions is 1,451 nt. However, this difference could be due to the longer length of REST 3’-UTRs (Fig 1A). In fact, the mean of the distance between multiple miRNA binding sites for TSH predictions in REST 3’-UTRs is 1,932 nt, which is very close to the value observed for REST pairs (Table 1).

We then computed the GC-content between multiple binding sites for the same family. The distributions of values are shown in Fig 2C. The average value is slightly lower for REST pairs than for TSH pairs (0.363 and 0.402, respectively; Table 1) and this is reflected in the distributions of values (orange and black curves, respectively; Fig 2C). This difference is not just due to the differences in GC content of the 3’-UTRs, since TSH pairs in REST 3’-UTR do have a higher average GC content of 0.391 (Table 1) and a distribution shifted to higher values (red curve; Fig 2C) compared to that of REST pairs. This confirms that lower GC content is a good predictor of miRNA binding sites, particularly between pairs of miRNA binding sites for the same family.

## 3. Discussion

Predictions of miRNA binding sites are not very accurate and are likely to include many false positives. Our hypothesis is that, given a subset of predictions assumed to be enriched in true positives, one could use it to compare its properties with those of the background of predictions to learn discriminant properties. For this strategy to have any chance of success, the selected subset needs to be significantly large. The largest databases that annotate experimentally supported target genes for human miRNAs, DIANA-TarBase v.8 and miRTarBase, overlap only about 10% [25]. Moreover, these databases contain indirect interactions that could originate from outside the 3’ UTR, with less strong evidence from high-throughput experiments, making these databases unsuitable candidates for our study.

In this work, we take advantage of an integrative approach that selects a large subset of predictions as more likely to be true because of their enrichment in transcription factor-regulated genes. This assumption is supported by the finding that the majority of microRNA families with validated high confidence interactions from miRTarBase are enriched in their targets with at least one specific transcription factor [26]. The redundancy in transcription factors and microRNA-associated targets could then be used to select for true interactions in biologically significant pathways [26], which highlights the potential of integrating network data for the selection of predictions with less false-positives.

Here we focused our analysis on miRNA families with targets enriched for REST targets. The transcription factor REST is a repressor that could be expected to have activity correlated to some miRNA families. Indeed, activity of miR-203 and REST co-regulate gene expression related to neuronal activity [27]. Additionally, miR-26 and miR-132 have been reported to target REST and its complex by participating in networks by negative feedback loops in neural tissue and controlling neurogenesis [28, 29].

Our strategy found 67 miRNA families with targets enriched in REST-regulated genes (S2 Table; see Methods and Materials for details). The targets of these 67 miRNA families in REST-regulated genes (S3 Table) constituted therefore our subset (REST pairs) to be compared to a background consisting of all other target predictions from TargetScanHuman 7.2 (TSH pairs).

Interestingly, REST pairs have more experimental support than TSH pairs (17.9% to 14.3%) according to DIANA-TarBase v.8 [30]; this difference increases when considering only the miRNAs reported in DIANA-TarBase v.8 (25.2% to 20.5%).

Since the 3’-UTR is the genetic region of the mRNA that contains cis-regulatory elements for miRNA regulation, we compared its length in the subset of putative REST-regulated genes with its length in all genes that have predicted miRNA binding sites, according to TargetScanHuman 7.2. We validated the prior observation that the 3’-UTRs of the set of REST-bound genes are significantly longer than those of the background [20] (Table 1; Fig 1A and 1B).

Position-specific analysis of miRNA binding sites in the 3’-UTRs of REST genes and TSH genes indicated that the predicted sites are located close to the 3’ and 5’ terminal ends, relative to the 3’-UTR length, more often than in the middle. Predictions for REST genes demonstrated a slightly higher density for the relative position of 0.8 and downstream (Fig 1H). Since for long 3’-UTR’s (>1300 nt), regions near the 3’-UTR terminals have been reported to carry more frequently conserved targeting sites [31], this finding is consistent with our assumption of a good selection of binding sites.

Moreover, we demonstrated that the subset of REST pairs has a continuous lower GC-content in the area surrounding the predicted target site, even when contrasting REST pairs with TSH pairs situated in REST 3’-UTRs. GC-poor, respectively AU-rich, 3’-UTR regions in close vicinity to miRNA binding sites have been described as correlating with target efficiency in multiple ways, such as by destabilizing mRNA and impeding the formation of stable and functional secondary structures, thus, providing accessible miRNA binding sites [31–33]. We conclude that the even lower GC content of REST pairs is consistent with our expectation that their predictions are more accurate than those of all TSH pairs.

Our results also revealed that REST pairs include significantly more target genes with multiple miRNA binding sites for a particular miRNA family than TSH pairs, as a proportion of the sum of target genes, suggesting that this feature is predictive of miRNA target sites. Multiple binding sites for the same miRNA might provide resistance against changes in the environment and accessibility, thus ensuring regulatory efficiency. The observation that 21 miRNA families, with the most predicted miRNA-mRNA interactions for REST pairs, display statistically significantly more target genes with multiple miRNA binding sites than the background hints at redundancy of the gene regulatory network and supports our assumption that our selection of transcription factor associated miRNA families and related predictions possess less frequent false positive predictions.

The analysis of GC-content between multiple miRNA binding sites provides another feature that separates REST pairs. We found lower GC-content between multiple miRNA binding sites for REST pairs than for TSH pairs and TSH predictions in REST 3’-UTRs (Fig 2C). Lower GC content might enable RNA-protein interaction by preventing stable secondary structure and this property can be taken as indicating good target predictions.

Our approach has revealed properties for miRNA families that are enriched in a subset of genes bound by the transcription factor REST, which indicate regulatory network interaction and clustered gene repression on the post-transcriptional level by miRNAs. To the best of our knowledge, our work represents the first attempt that selected large subsets of miRNA targets of different quality, based on the integration of miRNA-target relations with data from the network of transcriptional regulation, to collect features for the prediction of miRNA targets.

Our study has a number of limitations, including potential bias in the predictions used, and that our exploration used only ChIP-seq data regarding REST targets. Considering possible expansions of our approach, it is worth noting that we were able to provide a statistical assessment of significance given the relatively large number of genes targeted by REST. Using our approach with ChIP-seq or any other type of DNA-binding data for other transcriptional regulators will possibly only work for factors that regulate as many genes as REST does; these are not abundant. This means that extending the type of integrating method proposed here will need to add complexity, for example by pooling data for multiple factors and/or considering other indirect regulatory connections.

The reward of testing further network-based selections of miRNA targets is that our results could receive further support if the above-mentioned characteristics of GC-content and miRNA binding sites were detected for further transcription factors or network contexts. Network-based selection of miRNA-mRNA pairs can potentially provide further features to improve the algorithms used in miRNA prediction tools to ensure identification of conserved miRNA targeting.

## 4. Materials and methods

### 4.1 Datasets

#### 4.1.1 Human 3’-UTR sequences

TargetScanHuman 7.2 provides sequences for representative human 3’-UTRs based on GENCODE annotations with most 3P-seq tags. A total of 12,989 human 3’-UTRs were considered in the analysis.

#### 4.1.2 miRNA binding site predictions

miRNA binding site predictions for annotated human 3’-UTRs were obtained from TargetScanHuman 7.2. The predictions were based on finding complementary conserved mRNA sequences to the seed region of miRNAs (2–8 nt) and were ranked by the integration of further criteria [24]. To minimize biases in the predictions of TargetScan that could affect our analyses, we considered only predictions for broadly conserved miRNA families (conserved across most vertebrates) with miRNA targets conserved between human and mouse. The outcome comprises 219 broadly conserved miRNA families and 109,249 unique miRNA-target gene pairs for 120,702 predicted miRNA binding sites in human 3’-UTRs. TargetScanHuman 7.2 predictions and 3’ UTR sequences are publicly available and can be downloaded from http://www.targetscan.org/vert_72.

#### 4.1.3 REST target genes

We previously assigned target genes to the repressor REST by analyzing ChIP-seq datasets of 15 different cell types, including both neural and non-neural. In total 12,344 genes that are potentially regulated by REST were identified [20].

#### 4.1.4 Over-represented miRNA families for REST target genes

To determine the overlap between targets of miRNAs and transcription factors, we calculated the over-representation of broadly conserved miRNA families for the subset of REST-bound genes, from the background of all TargetScanHuman genes with predicted miRNA binding sites as previously described [20]. Briefly, for one ChIPseq dataset, given *n* REST-bound genes and *m* of them predicted to be target of a particular miRNA A, we randomly take *n* genes from the set of all genes with predicted TargetScan miRNA targets 10,000 times and count for the number of targets for miRNA A (*z*). To correct for the fact that REST-bound genes could have a higher tendency to have miRNA targets (e.g. due to longer 3’UTRs) we compute a factor (r) to correct z, which is the ratio between the number of all miRNA targets found in the *n* REST-bound genes and the number of all miRNA targets found in the random set of *n* genes. Then we multiply z by r to obtain the corrected value z*. This is repeated 10,000 times and we count how many times z* is smaller than m. The number of positive tests divided by the number of tests (10,000) is then taken as p-value of enrichment of miRNA A targets in the REST-bound genes. Computed p-values were corrected for multiple testing using the Benjamini and Hochberg method (S1 Table). The significance level for adjusted p-values was set to 0.05. The analysis resulted in 67 miRNA families with a number of predicted miRNA binding sites in the subset of potentially REST-regulated genes that was significantly higher than the background (S2 Table).

#### 4.1.5 Sets of miRNA-target gene pairs

For the purpose of studying the properties of miRNA binding sites in factor-bound genes, two sets of miRNA-target gene pairs were analyzed further. The first set comprised over-represented miRNA families for potentially REST-regulated genes that contain predicted miRNA binding sites according to TargetScanHuman 7.2; this includes 15,009 unique REST-associated miRNA-target gene pairs (REST pairs) (S3 Table). The second set covered 94,240 unique predictions for TargetScanHuman miRNA-target gene pairs (TargetScanHuman pairs), after excluding predictions of the first set from the background of all considered miRNA predictions listed in TargetScanHuman 7.2.

### 4.2 Statistics

In order to test statistical significance regarding the difference between the proportion of target genes with one and multiple predicted binding sites between TargetScanHuman pairs and REST pairs, p-values were calculated using the Fisher’s exact test [34].

**Fig 3:**
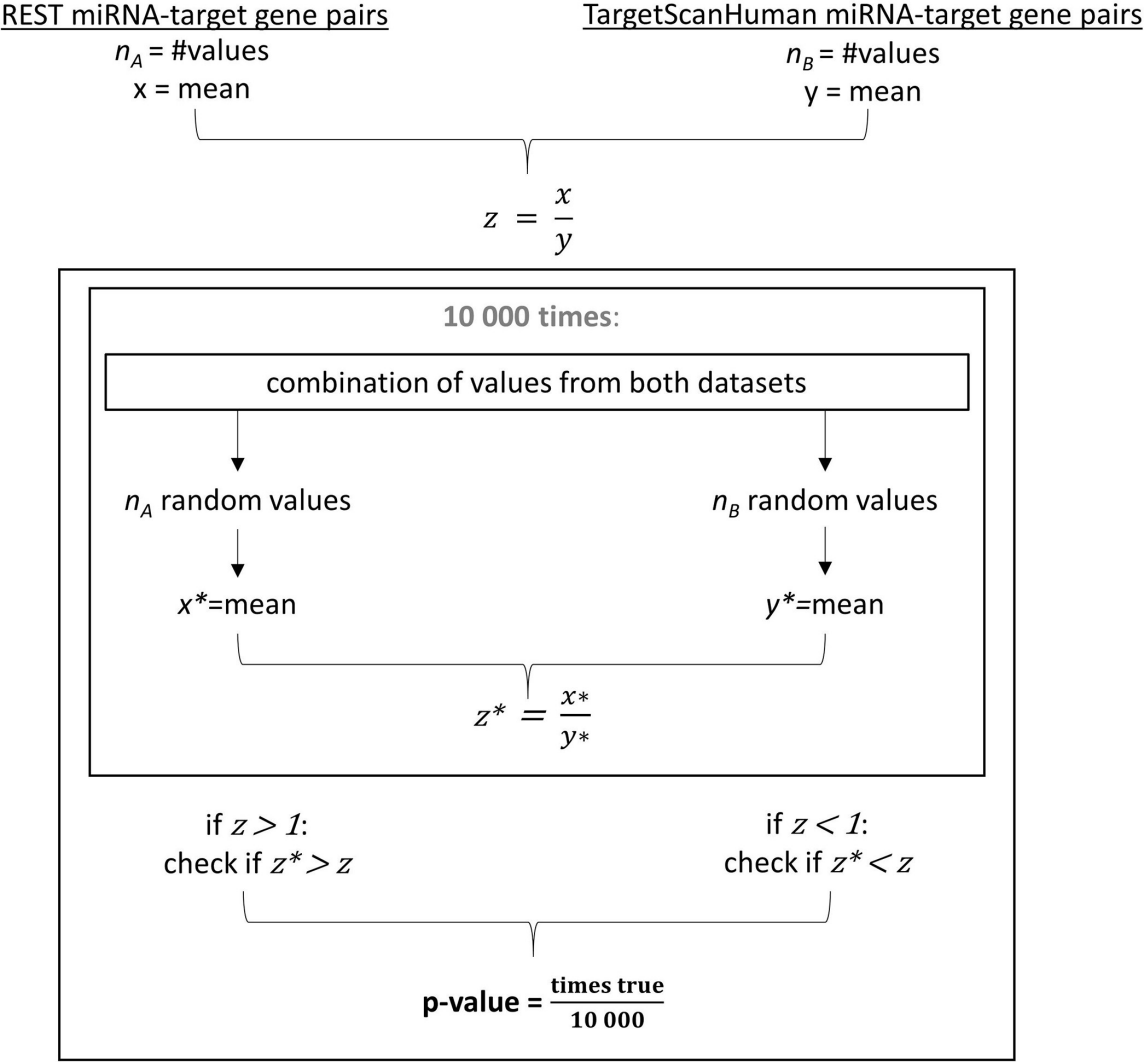
Illustration of p-value calculation by conducting 10,000 random tests for the statistical comparison of two sample means.

We also evaluated the significance of differences in several properties between REST miRNA-target gene pairs (REST pairs in REST-bound genes) and TargetScanHuman miRNA-target gene pairs (REST pairs in all genes considered in TargetScanHuman). These properties were distance from 3’UTR-start to miRNA binding site, distance from 3’UTR-end to miRNA binding site and relative position of the miRNA binding site in the 3’UTR. Statistical significance in terms of p-values was computed as described in the next paragraph (illustrated in Fig 3).

For each group of miRNA-target gene pairs to be compared (REST miRNA-target gene pairs and TargetScanHuman miRNA-target gene pairs), we obtained n_A_ and n_B_ values (for measured distances or GC-content), respectively. Means of the values (x and y, respectively) were calculated to produce the ratio value z = x / y. To produce random test ratios, we picked n_A_ and n_B_ values at random 10,000 times from the corresponding complete datasets of targets (all miRNA-targets in REST genes and all miRNA-targets in TargetScanHuman genes), without replacement. Means of the sampling values (x* and y*, respectively) were calculated to produce the random test ratio z* = x* / y*. Next, we examined whether the deviation from one was greater for z* than for z. For z > 1 we checked whether z*> z and took the number of successful tests divided by 10,000 as the p-value. In the case of z < 1 we defined a successful test as z*< z and calculated the p-value identically. The significance level was set to 0.05.

## Supporting information

S1 Table p-values and adjusted p-values for overrepresentation of REST target genes in miRNA family targets of fifteen cell types. Number of target genes for each miRNA in total background is shown.
(XLSX)

S2 Table miRNA families with predicted binding sites significantly enriched in the 3’-UTR of REST target genes (adjusted p-value < 0.05). Neural cell lines in grey. Values of “-”mean no significant enrichment (adjusted p-value > = 0.05).
(XLSX)

S3 Table Subset of TargetScanHuman predictions filtered for over-represented miRNA families and REST targets. (XLSX)

S4 Table Number of target genes with at least one and more than one predicted binding site, for a particular miRNA family. (XLSX)
